# Trends in educational inequalities in obesity‐attributable mortality in England and Wales, Finland, and Italy

**DOI:** 10.1002/oby.24225

**Published:** 2025-02-18

**Authors:** Fanny Janssen, Rolando Gonzales Martinez, Nicolás Zengarini, Pekka Martikainen, Anton Kunst

**Affiliations:** ^1^ Netherlands Interdisciplinary Demographic Institute‐Royal Netherlands Academy of Arts and Sciences/University of Groningen The Hague the Netherlands; ^2^ Population Research Centre, Faculty of Spatial Sciences University of Groningen Groningen the Netherlands; ^3^ Epidemiology Unit, ASL TO3, Piedmont Region Grugliasco Italy; ^4^ Helsinki Institute for Demography and Population Health, Faculty of Social Sciences University of Helsinki Helsinki Finland; ^5^ Max Planck Institute for Demographic Research Rostock Germany; ^6^ Max Planck – University of Helsinki Center for Social Inequalities in Population Health Helsinki Finland; ^7^ Department of Public and Occupational Health, Amsterdam University Medical Centre, University of Amsterdam Amsterdam the Netherlands

## Abstract

**Objective:**

We assessed trends in educational inequalities in obesity‐attributable mortality (OAM) and their contribution to educational inequalities in all‐cause mortality for people aged 30 years and older, in England and Wales (1991–2017), Finland (1978–2017), and Italy (1990–2018).

**Methods:**

In our population‐level study, we estimated the shares of all‐cause mortality due to OAM by educational level (i.e., low, middle, and high) by applying the population‐attributable fraction formula to harmonized obesity prevalence data by educational level, along with sex‐ and age‐specific relative risks of dying from obesity. We obtained OAM rates by multiplying the shares with individually linked all‐cause mortality data by educational level. We measured absolute inequalities in OAM and all‐cause mortality by the slope index of inequality.

**Results:**

OAM largely increased for the different sex‐ and education‐specific populations and increased most strongly for those with low educational level up to 2010 to 2015. Educational inequalities in OAM initially increased but stabilized or declined from at least 2008 onward. Obesity contributed, on average, 15% to absolute educational inequalities in all‐cause mortality in 1991 through 2017.

**Conclusions:**

The mortality impact of the obesity epidemic by educational level changed over time. Although the observed change from increasing to declining or stable educational inequalities is encouraging, reducing OAM in all socioeconomic groups remains a challenge.

**Study Importance**

**What is already known?**

- Although socioeconomic inequalities in obesity prevalence are well established, socioeconomic inequalities in obesity‐attributable mortality (OAM) are less widely studied, and little is known regarding their development over time.

**What does this study add?**

- Our study revealed a clear time dynamic in educational inequalities in OAM characterized by initial increases followed by declining or stable inequalities in more recent years.
- Across the studied populations, obesity contributed, on average, 11% to absolute educational inequalities in all‐cause mortality in 1991, compared with 13% in 2017.

**How might these results change the direction of research or the focus of clinical practice?**

- The observed change from increasing to declining or stable educational inequalities in OAM is encouraging. However, OAM is still a concern, particularly given the continued increases in OAM for males and females with middle educational level and for females with high educational level.
- There is still ample room and a continued need for actions to diminish obesity prevalence, to improve the level and access to care and treatment for obesity‐related diseases, and, consequently, to reduce OAM for all socioeconomic groups.

## INTRODUCTION

The health burden of the obesity epidemic differs by socioeconomic group [1, 2]. In high‐income countries, obesity prevalence and obesity‐attributable mortality (OAM) are currently higher among people with lower socioeconomic positions than among those with higher socioeconomic positions [1, 2]. However, this social gradient is not constant over time [3, 4]. Knowledge regarding levels and trends in the social gradient of OAM is highly relevant for obtaining a detailed picture of the mortality impact of the obesity epidemic across different socioeconomic groups, as well as potential changes therein over time. This is essential not only for monitoring purposes but also for appropriate targeting of preventive measures and health care policies. In addition, the changing social gradient of OAM likely has implications for levels and trends in socioeconomic inequalities in all‐cause mortality.

Previous studies have clearly established the existence of socioeconomic differences in obesity prevalence levels [1, 2]. In addition, previous studies have found that obesity can significantly affect mortality inequalities by socioeconomic status. The literature review by Petrovic et al. illustrated that physical activity and diet contributed, on average, 16% and 19%, respectively, to socioeconomic differences in all‐cause mortality in high‐income countries [5].

Hoffmann et al. [6] assessed the impact of educational inequalities in obesity prevalence on mortality and on educational mortality inequalities in 21 European populations around 2001 to 2006. They found that, if the obesity prevalence among the least‐educated groups was reduced to that of the highest‐educated groups in the same country, all‐cause mortality would be reduced for the low‐educated groups by 2.8% (males) and 4.9% (females), on average, and 6.3% (males) and 15.9% (females), on average, of the relative all‐cause mortality inequalities would be eliminated [6]. Using a similar method, Mackenbach et al. showed that obesity and overweight combined contributed, on average, 7.7% (males) and 11.7% (females) to the difference in partial life expectancy (ages 35–79 years) between the high‐ and the low‐educated groups in 15 European countries in 2010 to 2016 [7].

By contrast, much less is known regarding trends in educational inequalities in OAM and the changing contribution of obesity to socioeconomic mortality inequalities. It is, however, likely that these inequalities are subject to important time dynamics.

First, a time dynamic is inherent to any epidemic, and it is assumed that an initial unprecedented increase in obesity prevalence and related OAM is eventually followed by a decline [3, 8]. This assumption is backed up by evidence that obesity prevalence has stabilized or even declined since 2000 among children and adolescents in high‐income countries [9, 10], and that rates of increase in obesity prevalence decelerated between 1990 and 2016 among people aged 20 to 84 years in 18 European countries and the United States [11].

Second, the timing of the wave‐shape trends of the obesity epidemic likely differs across socioeconomic groups, in accordance with the theoretical framework of Jaacks et al. [3] based on their finding that socioeconomic inequalities in obesity prevalence vary among countries at different stages of the epidemic. In line with the diffusion of innovation theory [12], it may be assumed that both the uptake of obesity and preventive measures tend to be adopted first among individuals with high socioeconomic positions and then, eventually, spread to those with lower socioeconomic positions.

Third, the few existing cross‐national studies for Europe that focused on trends in obesity prevalence by educational level showed that increases in obesity prevalence between 1990 and 2010 tended to be larger among the low‐educated than the high‐educated groups in most European countries [13], and that obesity prevalence later stagnated among either the higher‐ or lower‐educated groups [4]. The review by Rokholm et al. of national studies of obesity prevalence trends since 1999 also found evidence of recent stagnating increases among higher socioeconomic groups in some European countries [9].

Our objective is to examine trends in educational inequalities in OAM, as well as their changing contributions to educational inequalities in all‐cause mortality, for people aged 30 years and older by sex in England and Wales (E&W), Finland, and Italy. For this purpose, we employ a population‐level study using the following: 1) meticulously harmonized obesity prevalence data by educational level, sex, age (i.e., 30–99 years), and adjacent calendar years; 2) individually linked mortality data by educational level; and 3) state‐of‐the‐art estimation and analytical techniques.

## METHODS

### Design and setting

In our population‐level study, we analyzed OAM by educational level for people aged 30 years and older in E&W (1991–2017), Finland (1978–2017), and Italy (i.e., Turin; 1990–2018). We studied people aged 30 years and older to ensure the validity of educational attainment as a measure of socioeconomic status. We selected the three countries based on the availability of long‐term secondary obesity prevalence and mortality data by educational level.

### Main measures

Our main outcome measures are standardized OAM fractions and rates by educational level and educational inequalities in both OAM and all‐cause mortality. All measures are stratified by country, sex, and year.

In line with previous research [14], we distinguished the following three educational attainment groups according to the International Standard Classification of Education (ISCED) 1997 [15]: low (i.e., no, preprimary, primary, and lower secondary education; ISCED‐1997 0‐2); middle (i.e., upper secondary and postsecondary nontertiary education; ISCED‐1997 3‐4); and high (i.e., tertiary education; ISCED‐1997 5‐6).

### Data

We used secondary country‐specific aggregate data for people aged 30 years and over from the early 1970s onward: 1) a meticulously built uniform database of smoothed obesity prevalence by educational level, sex, age, and adjacent calendar years for England, Finland, and Italy [16]; 2) age‐ and sex‐specific all‐cause relative risks (RRs) of dying from obesity from the DYNAmic MOdeling for Health Impact Assessment (DYNAMO‐HIA) project [17] obtained from Hoffmann et al. [6]; and 3) individually linked all‐cause mortality data by educational level, sex, and 5‐year age groups (i.e., 30–34, 35–39, …, 90‐94, and 95+ years) based on a 1% representative sample for E&W (Office for National Statistics [ONS] Longitudinal Study) [18, 19], for all official residents in Finland (Statistics Finland), and in Turin, Italy (Turin Longitudinal Study) [20]. The ONS Longitudinal Study contains individual‐level census information (1971–2011) for a 1% sample of the E&W population, linked with administrative data, i.e., births, deaths, and cancer registrations. The Turin Longitudinal Study is a census‐linked study that monitors the social and health status of residents of the northern Italian city of Turin from January 1971 to the present.

Inputs to the obesity prevalence database by educational level were drawn from the available data on obesity (body mass index [BMI] ≥ 30 kg/m^2^) from national health surveys from the 1970s onward derived from the Health Survey for England (HSE), the Finnish National Institute for Health and Welfare (THL), and the Italian National Institute of Statistics (ISTAT) and were meticulously harmonized [4]. These data were then consolidated into data without missing years and with similar age groups across time through interpolation across years and smoothing across ages. Finally, the two‐dimensional smoothing algorithm of Rizzi et al. [21] was applied to obtain smoothed obesity prevalence by country (*c*), educational level (*e*), sex (*s*), single year of age (i.e., 25–100 years), and adjacent calendar years (*y*) [16].

See supplementary data and methods in online Supporting Information for more information regarding the data used.

### OAM by educational level

In order to estimate OAM by educational level, we first applied the population‐attributable fraction (PAF) formula by Benichou [22] to age‐, sex‐, and education‐specific obesity prevalence data, as well as age‐ and sex‐specific all‐cause RRs of dying from obesity. A similar PAF approach has been used before to identify the potential of obesity for reducing educational inequalities in all‐cause mortality [6] and to assess the impact of obesity on life expectancy in the general population [23, 24]. More specifically, we obtained strata‐specific OAM fractions (OAMFs; the share of all‐cause mortality due to OAM) for England, Finland, and Italy by aggregating the prevalence data into 5‐year age groups (i.e., 30–34, 35–39, …, 90–94, and 95+ years) (*x*) and applying the formula :

$${\text{OAMF}}_{c,y,e,s,x}=\frac{p_{c,y,e,s,x}\;\left({\mathrm{RR}}_{s,x}-1\right)}{1+\left(p_{c,y,e,s,x}\;\left({\mathrm{RR}}_{s,x}-1\right)\right)}$$

The sex‐specific RRs by 5‐year age groups (*RR*_*s,x*_) were obtained by applying linear interpolation to the sex‐specific all‐cause RRs of dying from obesity by broader age groups (i.e., 30–44, 45–59, 60–69, 70–79 years) from Hoffmann et al. [6]. Second, OAM rates (OAMRs) by educational level were obtained by multiplying the strata‐specific OAMFs by strata‐specific all‐cause death numbers.

### Statistical analysis

In order to examine the trends over time in OAMFs and OAMRs by educational level, we obtained estimates across all ages (i.e., 30–95+ years) by applying direct standardization. We estimated standardized OAMFs (SOAMFs) by applying a country‐specific standard mortality schedule, based on all‐cause death counts for the general national population by 5‐year age groups for the year 2017, to the age‐specific OAMFs by country, year, sex, and educational group. We estimated standardized OAMRs (SOAMRs) by applying the 2013 revision of the European Standard Population [25] for people aged 30 years and older to the age‐specific OAMRs by country, year, sex, and educational group.

We examined trends over time in both absolute and relative educational inequalities in OAM (30+ years) by means of the slope index of inequality (SII) and the relative index of inequality (RII), respectively. We calculated the RII by applying a multiplicative Poisson regression model by year and sex, adjusted for age groups [26], to our OAM data. The SII was calculated from the RII and the SOAMRs in the general population [14]:

$${\mathrm{SII}}_{c,y,s}^{\mathrm{OAM}}=\frac{2*{\text{SOAMR}}_{c,y,s}*\left({\mathrm{RII}}_{c,y,s}^{\mathrm{OAM}}-1\right)}{\left({\mathrm{RII}}_{c,y,s}^{\mathrm{OAM}}+1\right)}$$

In order to assess the changing contributions of OAM to educational inequalities in all‐cause mortality, we estimated yearly potential declines in absolute educational inequalities in all‐cause mortality (measured by the SII) by eliminating OAM. We did so by subtracting the SII for non‐OAM (i.e., all‐cause mortality minus OAM) from the SII for all‐cause mortality. In order to obtain the relative contributions, we divided the yearly potential declines by the SII for all‐cause mortality.

We performed different sensitivity analyses, including the use of a different estimation of OAMRs and the use of different absolute inequality measures for all‐cause mortality and non‐OAM to assess the changing contributions of OAM to educational inequalities in all‐cause mortality (online Supporting Information). Their outcomes are discussed in the “Evaluation of data and methods” section (first sensitivity analysis) and the “Results” section (all other sensitivity analyses).

## RESULTS

**FIGURE 1:**
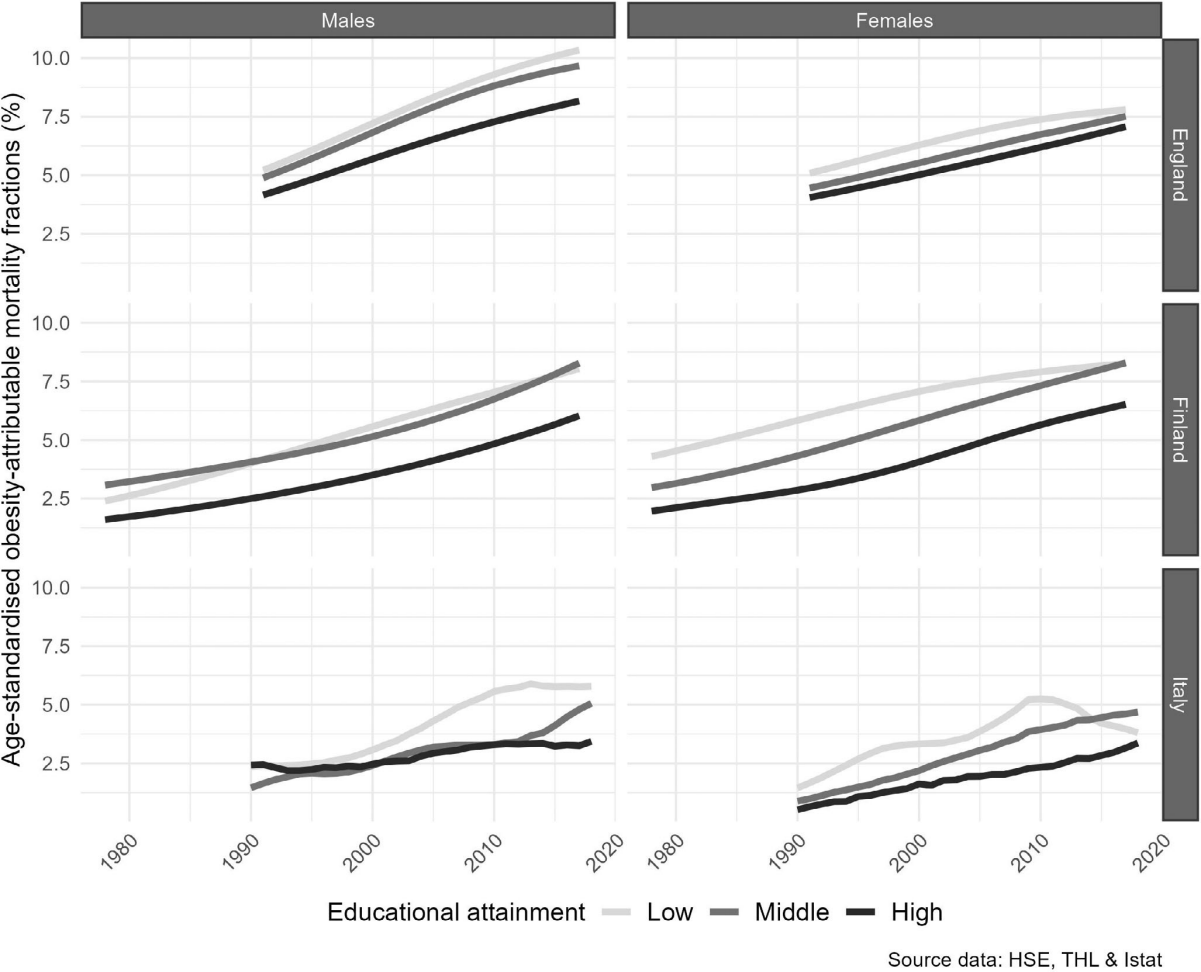
Trends in standardized obesity‐attributable mortality fractions (OAMFs) by educational level, for people aged 30 years and older in England (1991–2017), Finland (1978–2017), and Italy (1990–2018), by sex and country.

**FIGURE 2:**
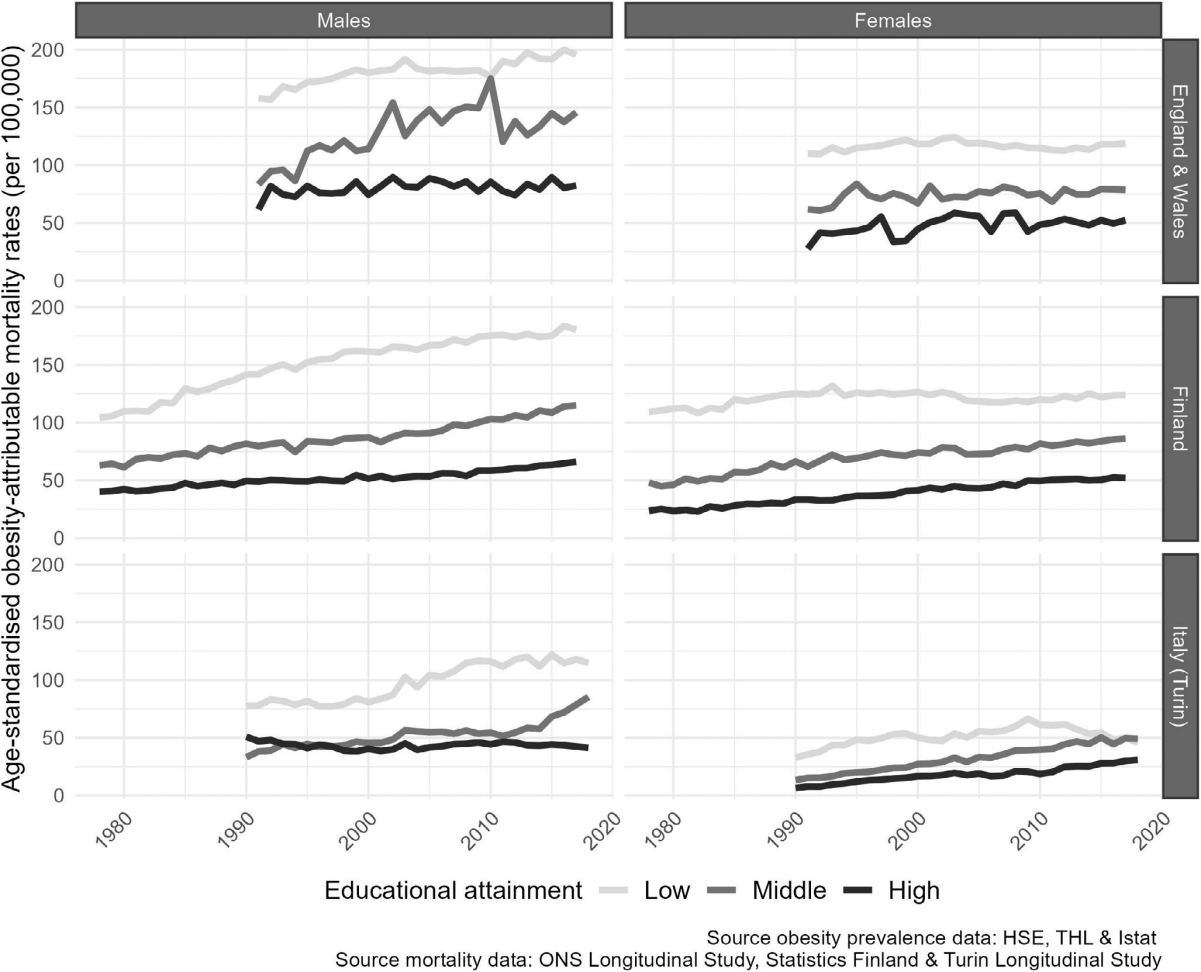
Trends in standardized obesity‐attributable mortality rates by educational level, for people aged 30 years and older in England and Wales (1991–2017), Finland (1978–2017), and Italy (Turin; 1990–2018), by sex and country.

SOAMFs and SOAMRs were higher for males than for females and showed a clear social gradient from 1991 onward, except among Italian (Turin) females and in Finland (fractions only) in recent years, where OAM levels for the low‐educated group were comparable with or lower than those for the middle‐educated group (Figures 1 and 2).

Up to about 2010 to 2015, SOAMFs and SOAMRs were largely increasing over time, except among high‐educated Italian males in the 1990s. SOAMFs were increasing more strongly than SOAMRs. The observed increases were, generally, largest among the low‐educated groups. In more recent years, and particularly among the low‐educated groups, the increasing trends tended to level off or reached stable levels (Italian males) or declined (Italian females). Also among high‐educated Italian males, there was a clear trend toward stagnation. Among the middle‐educated groups, OAM continued to increase in recent years, except among middle‐educated British males.

**FIGURE 3:**
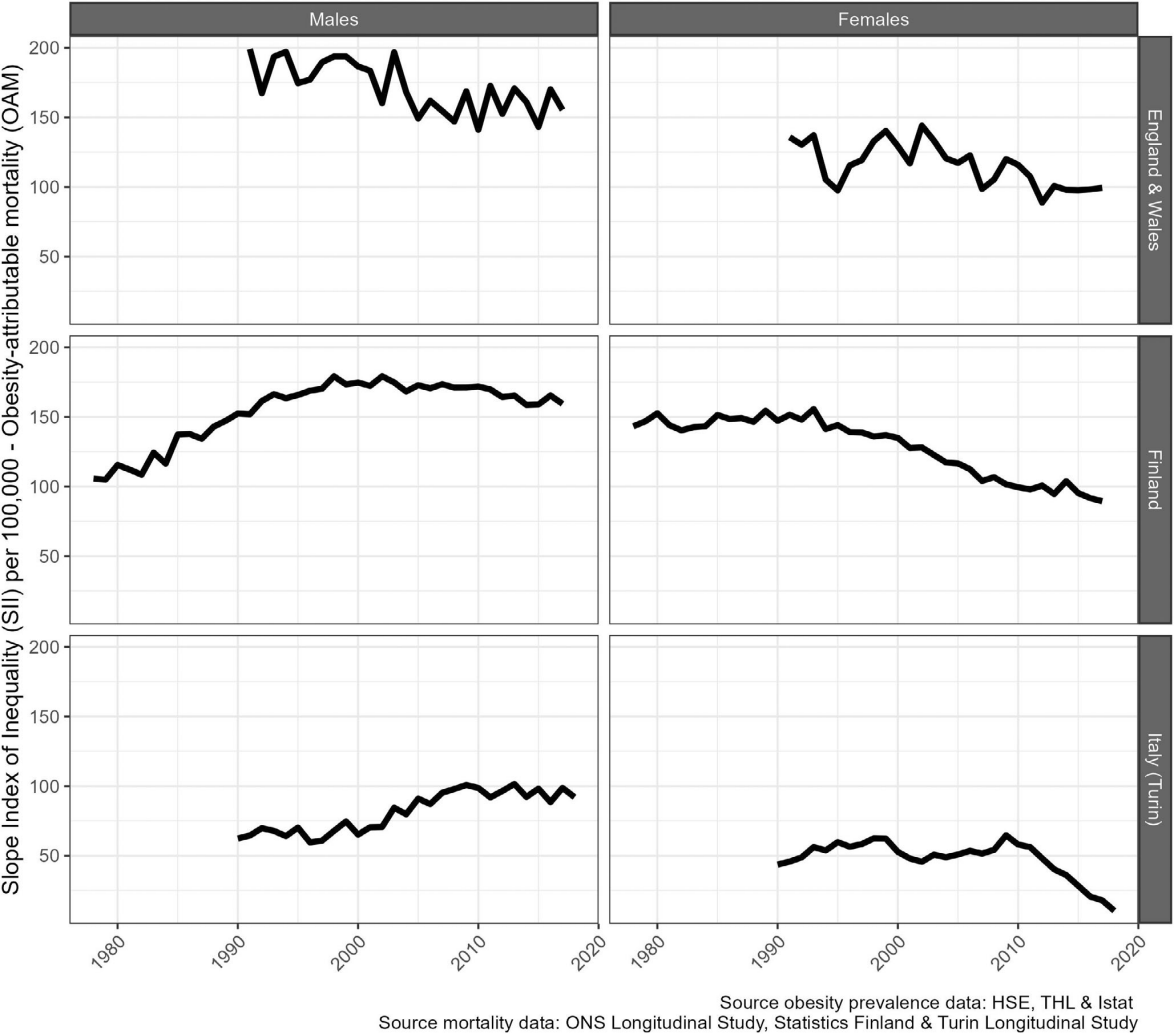
Trends in absolute educational inequalities in obesity‐attributable mortality, measured by the slope index of inequality, by sex and country, for people aged 30 years and older in England and Wales (1991–2017), Finland (1978–2017), and Italy (Turin; 1990–2018).

Absolute educational inequalities in OAM, measured by the SII, initially increased, except among British males, who experienced an initial decline (Figure 3). This initial trend was followed by a recent decline (females, Finnish males) or stabilization (British and Italian [Turin] males). The year in which the trend changed ranged from 1992 among Finnish females to 2008 among Italian males. The trend in relative educational inequalities in OAM, measured by the RII (Figure S1), followed a similar pattern, except that there was an initial decline among Italian females and a recent decline among Italian males.

**FIGURE 4:**
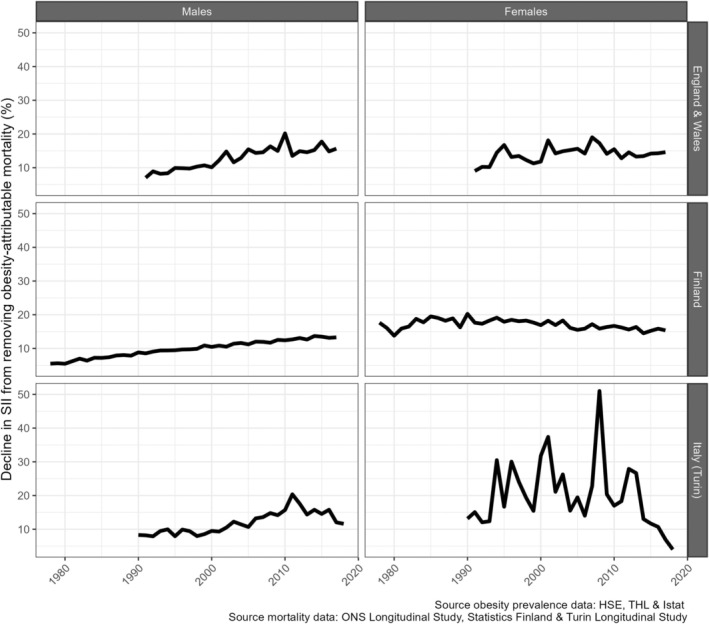
The (changing) relative contribution of obesity‐attributable mortality to absolute educational inequalities in all‐cause mortality (measured by the slope index of inequality [SII]), for people aged 30 years and older in England and Wales (1991–2017), Finland (1978–2017), and Italy (Turin; 1990–2018), by sex and country.

Obesity contributed, on average, 15% to absolute educational inequalities (measured by the SII) in all‐cause mortality for the six populations over the 1991‐2017 period (Table 1). This average contribution was largely similar when using the rate difference instead of the SII to measure educational inequalities in all‐cause mortality but was lower (at 11%) when the two different measures of absolute educational inequalities in remaining life expectancy at age 30 years were considered instead (Table S1). However, the contribution was not stable over time, as it generally increased initially and then declined or reached stable levels more recently, irrespective of the inequality measure applied (Figure 4; Figure S2). The turning point of the trend was around 2010, except among Finnish females (around 1990). In E&W and among Finnish males, increases were followed by stable levels, whereas in Italy (Turin) and among Finnish males, increases were followed by declines. In 1991, the contribution of obesity was, on average, 11% across the six populations. In 2017, the contribution was, on average, 13%, was slightly higher for males (13.7%) than for females (12.3%), and was highest in E&W (approximately 15%). The fluctuating levels for females in Italy (Turin) are due to low levels of both OAM (Figure 1) and educational inequalities in all‐cause mortality [27].

**TABLE 1:** Relative contribution of obesity‐attributable mortality (OAM) to absolute educational inequalities in all‐cause mortality (measured by the slope index of inequality [SII]) in 1991, 2017, and averaged over 1991 through 2017, for people aged 30 years and older, by sex and country.

Population	1991	2017	1991–2017
E&W, males	7.0%	15.6%	12.8%
E&W, females	9.0%	14.6%	14.0%
Finland, males	8.5%	13.3%	11.3%
Finland, females	17.6%	15.4%	16.9%
Italy (Turin), males	8.2%	12.0%	12.0%
Italy (Turin), females	15.1%	7.0%	21.0%
Average (unweighted)	10.9%	13.0%	14.7%
Average, males	7.9%	13.7%	12.1%
Average, females	13.9%	12.3%	17.3%

*Note*: Sources for obesity prevalence data by educational level were the HSE, THL, and ISTAT. Sources for mortality data by educational level were the ONS Longitudinal Study, Statistics Finland, and the Turin Longitudinal Study.

## DISCUSSION

### Summary of results

Standardized OAM shares and rates predominantly increased for the different sex‐ and education‐specific populations, most strongly for the low‐educated groups up to 2010 to 2015, and with larger increases in the shares of all‐cause mortality due to obesity than in OAM rates. More recently, increases in OAM stagnated among the low‐educated groups but continued among the middle‐educated groups and among high‐educated females. Educational inequalities in OAM initially increased in most studied populations but stabilized or declined from at least 2008 onward. Obesity contributed, on average, 15% to absolute educational inequalities (measured by the SII) in all‐cause mortality for males and females in the three populations over the 1991 through 2017 period. This contribution was 11% in 1991 and 13% in 2017.

### Evaluation of data and methods

In order to estimate OAM by educational level, we applied, similar to Hoffmann et al. [6], a PAF approach using obesity prevalence data and all‐cause mortality by educational level, as well as RRs of dying from obesity. This approach takes into account that obesity contributes to many different causes of death, and that its contribution to most of them is small [28]. Furthermore, the PAF approach relies strongly on the available data by educational level, which is an important advantage for studying socioeconomic inequalities in mortality. Because comparable cause‐specific RRs of dying from obesity for all the different obesity‐related causes were unavailable, the PAF approach could not be based on causes of death. Moreover, it should be noted that the PAF approach does not consider a time lag between obesity prevalence and OAM, and its estimates rely on the quality of the prevalence data and the specificity of the RRs that were used, discussed further subsequently.

We used obesity prevalence data by educational level, sex, and age for adjacent calendar years based on the application of interpolation and advanced smoothing techniques [16] to meticulously harmonize prevalence data based on data from national health surveys [4]. Because of important differences in the original survey data, the prevalence data for the three countries, and, consequently, any outcomes obtained from them, cannot be regarded as fully comparable among countries. First, the data were based on measured height and weight in England and on self‐reported height and weight in Finland and Italy. Second, educational attainment was classified according to the highest degree completed in England and Italy and to self‐reported school years attended in Finland. Third, the obesity prevalence data were unweighted for England and Finland but were weighted for Italy. In addition, the smoothed prevalences for Italy for 1991 through 1998 and for Finland for 1978 through 1992 should be treated with some caution. For Italy, the smoothed prevalences for 1991 through 1998 mainly relied on data for 1990, 1994, and 1999 only. For Finland, data on the older age groups (i.e., 65+ years) were missing up to 1992 and had to be extrapolated.

The RRs from Hoffmann et al. [6] came from a review of mainly Western European and American studies that controlled for potential confounders [17]. In the absence of comparable RRs across countries and over time, we applied one set of age‐ and sex‐specific RRs to the three countries and to all the years in our study, in line with previous research [6, 23]. Consequently, country differences and secular trends in the treatment of obesity‐related diseases were not considered. Moreover, in the absence of education‐specific RRs of dying from obesity, we applied, following Hoffmann et al. [6], similar RRs to the three educational groups. It is not yet known whether the impact of obesity can indeed be regarded as a (biological) constant or whether it differs among socioeconomic groups, e.g., because of a different interaction with other vulnerabilities and, if it differs, to what extent [6]. At present, the potential bias of using similar RRs for the three educational groups cannot be assessed.

In order to obtain OAM rates, we applied the OAM factors to all‐cause death counts for all of the different strata. Consequently, the trends over time in OAM rates might be influenced by the overall declining trends in all‐cause mortality [29]. This appears to explain why the standardized OAM rates showed less‐clear increases over time than the standardized OAM factors. Moreover, the education‐specific trends in OAM might be influenced by the substantial effects of other lifestyle factors, mainly smoking and alcohol use, on all‐cause mortality trends [24], as well as by differences in these effects by socioeconomic group. However, a sensitivity analysis in which we applied the OAM factors to non‐smoking plus non‐alcohol‐related mortality by stratum revealed largely similar trends in standardized OAM rates by educational level (Figure S3).

Whereas the obesity prevalence data referred to England and Italy, the mortality data referred to England and Wales and the Italian city of Turin. Because the Turin Longitudinal Study is considered to represent mortality and educational inequalities therein across Italy relatively well [30], we expect the OAM rates by educational level for Italy (Turin) to largely reflect the rates for Italy as a whole. Obesity prevalence and mortality rates, as well as educational inequalities therein, are likely to differ somewhat between Wales and England. However, because England's population is about 17 to 18 times larger than the Welsh population [31], any potential differences would have only a small effect on the estimates for England and Wales as a whole.

We focused on OAM and not on high BMI‐attributable mortality (obesity plus overweight). We advise against generalizing our outcomes to high BMI‐attributable mortality because of differences between obesity and overweight in terms of their prevalence, socioeconomic gradient, and association with mortality. Moreover, because the association with mortality is less well established for overweight than for obesity, and overweight is not always significantly associated with increased mortality [32], our outcomes should not simply be regarded as conservative estimates of the contribution of above ideal weight.

We used aggregate secondary obesity prevalence and all‐cause mortality data by country, year, educational attainment, sex, and 5‐year age group. A further layer of detail, e.g., migrant group or marital status, would undoubtedly be interesting but would result in too many strata with very small numbers. Our results are therefore restricted to educational inequalities. Further studies on other domains of inequality are urgently needed.

### Explanation of results

The observed social gradient in OAM contributed, on average, 15% to absolute educational inequalities in all‐cause mortality for males and females in the three studied populations over the 1991 through 2017 period. This contribution lies in between the 12% and 21% that Petrovic et al. [5] reported for the average contribution of physical activity and diet, respectively, to educational differences in all‐cause mortality between 1948 and 2016 in predominantly high‐income countries. Our average contribution is also close to the average contribution of 13% that Hoffmann et al. [6] found for our studied populations in 2001 through 2006 (Table S2). Despite some differences in the results for individual countries, which are likely due to data and methodological differences (see the note below Table S2), both their and our results point to substantial contributions of obesity to educational inequalities in mortality. These contributions appear to be slightly larger than the contributions of obesity to mortality in general in Europe (approximately 9% to 11%) [4, 5, 6], which confirms the importance of the educational gradient in obesity. To our knowledge, our study is the first to document that the contributions of obesity to educational inequalities in mortality and life expectancy initially increased and declined or reached stable levels more recently.

Our finding that educational inequalities in OAM initially increased in most studied populations can be related to the observation that the low‐educated groups generally exhibited the largest increases in age‐standardized OAM up to about 2010 to 2015. These findings correspond to Stage 3 of the obesity transition theory by Jaacks et al. [3], which posits an acceleration of increases in obesity among people with lower socioeconomic status. Indeed, the trends in the underlying age‐standardized obesity prevalence by educational level (Figure S4) also showed that increases in obesity prevalence tended to be larger among the low‐educated groups than among the high‐educated groups, which is in line with previous findings [13]. However, these trends cannot be readily interpreted in terms of the diffusion of innovation theory by Rogers [12] because the educational differences in obesity prevalence trends were small and because the obesity prevalence levels among the high‐educated groups in the early years were too low to have facilitated diffusion [4]. Instead, it may be assumed that environmental changes resulted in decreased physical activity (i.e., occupational and transportation‐related) and changed diet patterns [33] for all members of society [4], albeit with a greater impact for the low‐educated groups.

Our observation that educational inequalities in OAM declined or stabilized more recently can be related to the more favorable trends in OAM among the low‐educated groups, coupled with (continued) increases in OAM among the middle‐educated groups and increasing (for females) or stable (for males ) OAM levels in recent years among the high‐educated groups (Figure 2). The recent trends in obesity prevalence by educational level also showed stagnating increases among the low‐educated groups in England and Italy and among the high‐educated groups in Italy (Figure S4). Stagnating trends in obesity prevalence among higher socioeconomic groups in selected European countries have been reported before [4, 9] and, when they have occurred before stagnating trends among the low‐ and middle‐educated groups (such as for Italian males), are considered in line with the assumed diffusion of preventive measures from individuals with higher socioeconomic positions to those with lower socioeconomic positions [4]. However, our finding that obesity prevalence stagnated predominantly among the low‐educated groups in England, which was also observed by Kagenaar et al. [4], is not in line with this theory. Instead, this trend might be the result of the implementation of obesity‐related policies that are either more likely to benefit the lower‐educated [4], such as tax‐related fiscal interventions [34], or are particularly addressed to the low‐educated groups because of their already high levels of obesity prevalence and OAM [11, 23]. In this context, the sugar tax on soft drinks that the UK government introduced in 2016 [35] might have played an important role. More generally, the country and sex differences in the education‐specific occurrence and timing of the recent stagnation in obesity prevalence and OAM likely depend, at least partly, on the focus (i.e., population‐wide or targeted) and population‐specific impact of national prevention policies.

## CONCLUSION

The mortality impact of the obesity epidemic by educational level, and, consequently, educational inequalities in OAM, clearly changed over time in the three high‐income European populations studied. The general pattern of initial increases followed by declining or stable educational inequalities in OAM was largely driven by the low‐educated groups initially exhibiting the largest increases in obesity prevalence and, consequently, OAM, followed by more favorable trends in recent years. However, the observed change from increasing to declining or stable educational inequalities in OAM is not only cause for optimism, as OAM continued to increase for the middle‐educated groups (males and females) and for high‐educated females. In addition, OAM remained higher among the low‐educated groups than among the other educational groups. Moreover, the inequalities in OAM continued to contribute substantially to all‐cause mortality inequalities. Therefore, there is still ample room and a continued need for actions to diminish obesity prevalence, to improve the level and access to care and treatment for obesity‐related diseases, and, consequently, to reduce OAM for all socioeconomic groups.

## AUTHOR CONTRIBUTIONS

Fanny Janssen conceptualized the study and acquired the data. Pekka Martikainen arranged access to the Finnish mortality data by educational level, and Nicolás Zengarini provided the mortality data by educational level for Italy (Turin). Fanny Janssen and Rolando Gonzales Martinez performed the data handling. Rolando Gonzales Martinez developed the obesity prevalence database and estimated obesity‐attributable mortality by educational level. Fanny Janssen conducted further analyses and drafted the manuscript. Anton Kunst and Pekka Martikainen revised the draft critically for intellectual content. All authors approved the final submitted version of the work.

## FUNDING INFORMATION

This work is funded by the Netherlands Organization for Scientific Research (NWO) in relation to the research project “Forecasting future socioeconomic inequalities in longevity: the impact of lifestyle ‘epidemics,’” under grant no. VI.C.191.019 (https://www.futurelongevitybyeducation.com/). In addition, Pekka Martikainen was supported by the European Research Council under the European Union's Horizon 2020 research and innovation programme (grant agreement no. 101019329); the Strategic Research Council (SRC) within the Academy of Finland grants for ACElife (#352543‐352572) and LIFECON (# 345219); and grants to the Max Planck ‐ University of Helsinki Center from the Jane and Aatos Erkko Foundation (#210046), the Max Planck Society (# 5714240218), University of Helsinki (#77204227), and the cities of Helsinki, Vantaa, and Espoo. The funding sources had no role in the study design, collection, analysis, or interpretation of the data; in the writing of the manuscript; or in the decision to submit the paper for publication. The study does not necessarily reflect the views of the funding organizations, and in no way anticipates the future policy in this area of the funding organizations.

## CONFLICT OF INTEREST STATEMENT

The authors declared no conflicts of interest.

## Supporting information


**Supplementary File S1:** Supplementary Data and Methods.

**Supplementary File S2:** Supplementary Tables and Figures.

## Data Availability

The secondary data that we used as input for our analyses cannot be made publicly available, because the institutes that own the data apply a restricted access policy. Access to these data can, however, be requested through the institutes that own the data. The specific output based on aggregate data that we used to create the main figures can be obtained through Open Science Framework (https://osf.io/795q3/). The R code(s) can be requested from the corresponding author.
