# Supplementary material for: Trends in educational inequalities in obesity‐attributable mortality in England and Wales, Finland, and Italy
Source: Obesity (Silver Spring). 2025 Feb 18;33(3):578–88. doi: 10.1002/oby.24225 (PMC11897850; doi:10.1002/oby.24225)
Supplement: Supplementary file 1 — Supplementary File S1: Supplementary Data and Methods. [file OBY-33-578-s001.pdf]

TRENDS IN EDUCATIONAL INEQUALITIES IN OBESITY-ATTRIBUTABLE MORTALITY IN ENGLAND & WALES, FINLAND, AND ITALY - SUPPLEMENTARY FILE 1 – SUPPLEMENTARY DATA AND METHODS

ADDITIONAL INFORMATION REGARDING THE DATA

- Summary of the data used

To estimate obesity-attributable mortality by educational level for England & Wales (1991-2017), Finland (1978-2017), and Italy (Turin)(1990-2018), we used obesity prevalence data by educational level, all-cause relative risks of dying from obesity, and all-cause mortality and population data by educational level. Table A1 summarizes the data sources and the years to which the data apply. In the remainder of this document we will describe the data in more detail.

Table A1. Summary information on the data sources used, the follow-up designs applied, and the resulting aggregate, by country

	England &Wales	Finland	Italy (Turin)
**Obesity prevalence data by educational level, sex and age (25+)**	1991-2018 (England)	1978-2020	1990-2018 (Italy)
**Source smoothed obesity prevalence data**	Gonzales Martinez and Janssen, 2023		
**Underlying sources prevalence data**	National Health Service (NHS): HSE	Finnish Institute for Health and Welfare (THL): AVTK, EVTK, ATH, FINSOTE	Italian National Institute of Statistics (ISTAT): NMSS, HCHS, AVQ
**All-cause mortality & population data by educational level, sex and age (30+)**	1972-2017	1971-2017	1972-2019
**Source of mortality data**	ONS Longitudinal Study	Statistics Finland	Turin Longitudinal Study
**Obesity-attributable mortality data by educational level, sex and age (30+)**	1991-2017	1978-2017	1990-2018

HSE: Health Survey for England; AVTK: Health Behaviour and Health among the Finnish Adult Population; EVTK: Health Behaviour and Health among the Finnish Retirement-Age Population; ATH: Adult Health, Welfare and Service Research; FinSote: FinSote National Survey of Health; NMSS: National Multipurpose Social Survey; HCHS: Health and Healthcare Utilisation Study; AVQ: Aspects of Daily Life

- Obesity prevalence data by educational level

We used a meticulously built uniform database of obesity prevalence for England, Finland, and Italy by country, educational level (low, middle, high)(ISCED 1997)(UNESCO, 1997), sex, single year of age (25-100), and adjacent single calendar years (Gonzales Martinez and Janssen, 2023). The inputs are the data from available national health surveys from the 1970s onwards, which were derived from the Health Survey for England (HSE), the Finnish National Institute for Health and Welfare (THL), and the Italian National Institute of Statistics (ISTAT), and were meticulously harmonized (Kagenaar et al. 2022). Subsequently, these data were consolidated into data without missing years and with similar age groups across time, by interpolation across years and smoothing across ages. Finally, the two-dimensional smoothing algorithm of Rizzi et al. (2019) was applied to obtain smooth trends over time and smooth patterns across ages.

Table A2 details the national health surveys and their data, which served as the underlying obesity prevalence data by educational level in England, Finland, and Italy. Appendix A1 details the underlying data sources. There are some important differences between the three countries in the obesity prevalence data by educational level. First, the data were based on measured height and weight in England, and on self-reported height and weight in Finland and Italy. Second, educational attainment was classified according to the highest degree completed in England and Italy, and to self-reported school years attended in Finland. Third, the obesity prevalence data were unweighted for England and Finland but were weighted for Italy. In addition, the data were grouped in dissimilar age groups, and strata with missing data existed because of low cell counts. Details regarding the harmonization of these data, including the conversion of the country-specific educational classification to the ISCED 1997 classification, can be found in Supplementary File II of Kagenaar et al. (2022).

However, the harmonized data still presented some important challenges that needed to be overcome to build a uniform database. Whereas data from a single survey could be used for England, data from multiple surveys had to be combined for Finland and Italy. Yearly prevalence data by educational level were available for England and Finland, whereas the prevalence data between 1990 and 2000 for Italy essentially relied on data for three survey years only (1990/91, 1994, 1999/2000). For Finland, data regarding the older age groups (65-84) were missing for the years up to 1992, and were available only bi-annually from 1993 to 2013.

Table A2. The national health surveys and their data used to build the database on obesity prevalence by educational level, sex and age (25+), for England, Finland, and Italy

	England (1991-2018)	Finland (1978-2020)	Italy (1990-2018)
**Surveys**	HSE (1991-2018)	AVTK (1978-2014); EVTK (1993-2013, bi-annually); ATH (2015-2017); FinSote (2018-2020)	NMSS (1990/1991); HCHS (1994, 1999/2000); AVQ (2001-2018)*
**Sample size**	7658 ~ 22619	2520 ~ 5110; 1673 ~ 1882; 2305 ~ 49846; 6134 ~ 26371	65264 ~ 67400; 62461 ~ 128039; 44682 ~ 55294
**Age range**	15-85+	15-64; 65-84; 20-90+; 10-90+	18-80+; 15-85+; 18-75+
**Age interval**	5 years (1991-2013, 2015-2018) 10 years (2014)	10 years	5 years (1990, 1994, 1999, 2001-2012) 10 years (2013-2018)
**Data (height; weight)**	Measured	Self-reported	Self-reported
**Data (educational level)**	Highest attained level of education	Years of schooling	Highest attained level of education
**Data (weighting)**	Unweighted	Unweighted	Weighted

HSE: Health Survey for England; AVTK: Health Behavior and Health of Adult Population; ATH: Adult Health, Welfare and Service Research; FinSote: FinSote National Survey of Health; NMSS: National Multipurpose Social Survey; HCHS: Health Conditions and use of Health Services; AVQ: Aspects of Daily Life.

* We applied the NMSS data (1990/1991) to 1990, and the HCHS data for 1999/2000 to 1999.

Gonzales Martinez and Janssen (2023) dealt with these obstacles in two steps. First, the data were consolidated into data without missing calendar years and with similar five-year age groups across time. Second, two-dimensional smoothing using the Rizzi et al. (2019) algorithm was applied to obtain both smooth trends over time and smooth patterns across ages.

The consolidation step included linear interpolation across age groups and linear interpolation across years to obtain obesity counts for age groups with missing data and for calendar years with no data, respectively. This was done by averaging the information of the previous and subsequent age group or the previous and subsequent survey year. For Finland, the missing information for the elderly was predominantly obtained through extrapolation. In addition, to deal with dissimilar age groups over time (size, start of open-ended age interval), one-dimensional smoothing (Rizzi et al. 2015) across age groups was applied. In doing so, we used the ungroup package in R (Pascariu et al., 2018), and selected everywhere age 100 as the upper limit. Finally, the smoothed death counts by single year of age were aggregated into either five- or 10-year age groups, in line with the additional country-specific data.

For England, in the absence of missing data, no interpolation or extrapolation was applied to the data. To obtain the data for 2014 (originally in 10-year age groups) in five-year age groups, we applied one-dimensional smoothing of the counts over age, and subsequently aggregated the counts into five-year age groups. The consolidated data for England, consequently, have yearly information from 1991 to 2018, with uniform five-year age groups (25-29, …, 75-79, 80+).

For Finland, we first applied linear interpolation to deal with occasionally missing strata. In addition, we linearly interpolated the missing information for those aged 65-84 in 1994, 1996, …., 2012, 2014 based on the age-specific information for those aged 65-84 for the year before and after. To deal with the missing information for those aged 65-84 in 1978-1992, we extrapolated backwards – by linear regression for each age group – the age-specific information available between 1993 and 2013 (1993, 1995, … , 2011, 2013). Finally, 1D smoothing was applied to the data from 1978 to 2014 (15-84; 10-year age groups), which resulted in data by single year of age from age 15 to age 99, which we subsequently aggregated into 10-year age groups, in line with the data for the later years. The consolidated data for Finland, consequently, have yearly information from 1978 to 2020, with uniform 10-year age groups (20-29, …, 80-89, 90+).

For Italy, to obtain information for the calendar years with no data (1991-1993, 1995-1998, 2000), we interpolated both the obesity counts and the population counts on the basis of the available information for adjacent years. Specifically, the counts in 1990 and 1994 were interpolated to obtain an estimate of the counts (up to 80+) from 1991 to 1993; the counts in 1994 and 1999 were interpolated to obtain estimates of the counts (up to 85+) from 1995 to 1998; and the counts in 1999 and 2001 were interpolated (up to 75+) to obtain an estimate of the counts in the year 2000. Additionally, 1D smoothing was applied to the information for all the years from 1990 to 2013 to obtain uniform age groups (20-85+, five-year age intervals). The consolidated data for Italy, consequently, have yearly information from 1990 to 2018, with uniform five-year age groups (25-29, …, 80-84, 85+).

In the final step, two-dimensional (2D) smoothing with the algorithm by Rizzi et al. (2019) was applied to the consolidated prevalence data for Finland, England, and Italy. The 2D Rizzi et al. (2019) smoothing algorithm produces results that are robust to the presence of outliers, and is based on a bivariate Poisson

stochastic process, in line with obesity having a bivariate distribution of counts by age and calendar year. The Rizzi et al. (2019) algorithm maximizes a penalized likelihood of B-splines applied to the bivariate distributions of obesity by age and calendar year. Through this maximization, the Rizzi et al. (2019) algorithm produces detailed smooth surfaces of prevalence based on prevalence data of adjacent calendar years and age groups without missing strata. In the 2D smoothing algorithm, the optimization is based on the minimization of the Bayesian Information Criterion (BIC).

Table A3 shows the values of the hyperparameters we used in the 2D smoothing algorithms for each country. A pseudo-out-of-sample extrapolation experiment was performed to calibrate the hyperparameters of the 2D Rizzi algorithm based on obesity data for Finland. That is, the data by educational level for Finland for the years 2013 to 2020 were divided into one subsample with data comprising those aged 25-79 (the train sample) and another dataset comprising those aged 80-90+ (the test sample). The estimates of obesity prevalence by educational level for those aged 80 and older obtained through the train sample – using different experiments – were compared with the real prevalence for those aged 80 and older that was held out (the test sample). We subsequently tested the accuracy of the fit using different artificial end points versus burning the data beyond 100 years, different numbers of knots in the splines (from three to eight), and different values of the polynomial degree in the splines (from two to 12). The results showed that the most accurate fit is obtained by using a higher number or knot (equal to eight), a polynomial of degree equal to six, and a burn-in without endpoint imputation that discards smoothed values above 100 years. These choices were subsequently used when applying the two-dimensional Rizzi et al. smoothing for Finland, Italy, and England. However, for Italy, these choices did not result in a good fit, and it proved necessary to use different parameters to calibrate the 2D algorithm by sex and educational level.

Table A3. Values for the hyper-parameters used when two-dimensionally smoothing the obesity prevalence, by country, educational level, and sex

		Males			Females		
Country	Parameters	Low educated	Middle educated	High educated	Low educated	Middle educated	High educated
England	Knots of the spline	8	8	8	8	8	8
	Polynomial degree	6	6	6	6	6	6
	Last interval (years)	20	20	20	20	20	20
Finland	Knots of the spline	8	8	8	8	8	8
	Polynomial degree	6	6	6	6	6	6
	Last interval (years)	25	25	25	25	25	25
Italy	Knots of the spline	10	10	7	10	6	10
	Polynomial degree	4	4	3	4	3	8
	Last interval (years)	35	35	35	30	35	35

In the end, the database contains obesity prevalence data by educational level, sex, single year of age (25-100), and adjacent years for England (1991-2018), Finland (1978-2020), and Italy (1990-2018).
For the current analysis, the data were converted to prevalence data by five-year age groups (30-34, …, 90-94, 95+) by obtaining weighted averages using the single-age prevalence with the single-age population numbers by sex, educational level, country, and year, which we obtained through our longitudinal mortality follow-ups (see the final section of this document).

- **Relative risks (RR) of dying from obesity**

For the relative risks of dying from all-cause mortality associated with obesity, we retrieved the RRs from Hoffmann et al. (2015), by sex and broad age group (30-44, 45-59, 60-69, 70-79). These stem from the Dynamo-HIA project (Lobstein & Leach, 2010; Lhachimi et al., 2012) and from a review of studies mainly conducted in Western Europe and the USA that controlled for potential confounders, and studied either self-reported obesity or measured obesity or both. The RRs (approximately 1.55 for males and 1.50 for females) and the age pattern (slightly higher RRs at younger than at older ages) (see Table A4) were largely in line with the overall RR for obesity (1.64) estimated by the Global BMI collaboration (2016) and the differences across age groups found in the same study.

Because the data were only available in broad age groups, we applied linear interpolation to obtain the RRs by five-year age groups, in line with previous research (Vidra et al. 2019). Sensitivity analysis revealed that largely similar estimates of obesity-attributable mortality are obtained using either a linear or a quadratic interpolation (Gonzales Martinez & Janssen 2024). The interpolated RRs can be viewed in Table A4 below.

Table A4. Relative risks of dying (all-cause mortality) from obesity by age and sex, before and after applying the linear interpolation

	Original RR (Hoffmann et al. 2015)			Linearly interpolated RR	
**Age group**	**Males**	**Females**	**Age**	**Males**	**Females**
30-44	1.550	1.500	30-34	1.558	1.508
			35-39	1.552	1.503
			40-44	1.546	1.497
45-59	1.540	1.490	45-49	1.540	1.491
			50-54	1.534	1.486
			55-59	1.528	1.480
60-69	1.520	1.480	60-64	1.522	1.475
			65-69	1.516	1.469
70-79	1.500	1.450	70-74	1.510	1.463
			75-79	1.504	1.458
			80-84	1.498	1.452
			85-89	1.492	1.447
			90-94	1.486	1.441
			95-99	1.479	1.436

- **All-cause mortality data by educational level**

We used individually linked all-cause and cause-specific mortality data by highest educational attainment (low, middle, high), sex, five-year age group (30-34, …, 90-94, 95+), and single calendar year for England & Wales (1972-2017), Finland (1971-2017), and Italy (Turin) (1972-2019). These data stem from longitudinal mortality follow-ups in which individual data on mortality are linked to information on their educational attainment and the population at risk either five (Finland) or 10 years earlier (E&W; Italy (Turin)).

For Finland, the data stem from Statistics Finland and cover all official residents in Finland. For Italy, the data stem from the Turin Longitudinal Study (TLS), which is a census-linked study that monitors the social and health status of residents of the northern Italian city of Turin from January 1971 to the present (Costa and Demaria, 1988; Creeser 2001). The TLS is a longitudinal study based on the historical population register built to monitor metropolitan health variations linking the social and health careers of individuals and families. The TLS currently includes records for 2,391,833 persons who were resident in the city once or more since January 1971 (continually updated twice a year). The record, built through deterministic individual record-linkage procedures, includes the socio-economic information collected by the population censuses (1971, 1981, 1991, 2001, and 2011) and the health information derived from several statistical and administrative sources with different windows of follow up: causes of death (1971), hospital admissions (1995), diabetes registry (2002), cancer registry (1995), diagnostic tests or specialist visits (2000), drug prescriptions archives (1997), and screening programmes (2011). For England & Wales, we used data from the Office for National Statistics Longitudinal Study (ONS Longitudinal Study) (Office for National Statistics 2019), which contains individual information on demographic and socio-economic variables obtained through the censuses (1971, 1981, 1991, 2001, 2011), linked with individual information on life events, including births, deaths, and cancer registrations, for an approximately 1% representative sample of the population of England & Wales (Shelton et al. 2019). We adjusted the data for England & Wales to address data issues regarding the allocation of people to educational groups that hampered the study and international comparison of educational inequalities in mortality (Janssen et al. 2024).

See Table A5 for summary information regarding the used all-cause mortality data by educational level, by country. On https://www.futurelongevitybyeducation.com/background-information/ (Password = VICI_info) more information on the used all-cause mortality data by educational level can be found.

Table A5. Summary information regarding the all-cause mortality data by educational level, by country

	**England &Wales**	**Finland**	**Italy (Turin)**
**Source of data**	ONS Longitudinal Study	Statistics Finland	Turin Longitudinal Study
**Follow-up population**	Sub-sample (1%) of the E&W population aged 20 and older	Finnish population aged 25 and older	Turin population aged 20 and older covered in the population censuses
**Length of follow-up**	10 years	5 years	10 years
**(Underlying) source of educational data**	The different censuses (1971, 1981, 1991, 2001, 2011)	Registers of post-compulsory education certificates (Jan 1 1971, 1976, …. , 2016)	The different censuses (1971, 1981, 1991, 2001, 2011)
**Treatment of immigration**	Only considered when they were present at the start of the follow-up period		
**Treatment of emigration**	Individuals who emigrated and did not return during a given follow-up period were excluded from that follow-up period.	Individuals who emigrated and did not return during a given follow-up period were excluded from that follow-up period.	For individuals who emigrated and did not return during a given follow-up period, the person-time until their emigration date was counted.
**Treatment of temporal emigration**	For individuals who emigrated and came back within the same follow-up period, the person-time was counted for the full follow-up period.		
**Resulting data years**	1972-2017	1971-2017	1972-2019
**N of deaths** (30+; across the observation period)	257,413 (original); 247,425 (adjusted)	2,191,480	419,637
**N of personyears** (30+; across the observation period)	14,372,539 (original); 14,358,549 (adjusted)	142,033,351	27,665,912

Source data: ONS Longitudinal Study, Statistics Finland & Turin Longitudinal Study

ADDITIONAL INFORMATION REGARDING THE SENSITIVITY ANALYSES

We performed different sensitivity analyses, among which the use of a different estimation of obesity-attributable mortality rates, and the use of different absolute inequality measures for all-cause and non-obesity-attributable mortality to assess the changing contributions of OAM to educational inequalities in all-cause mortality.

The alternative estimation of obesity-attributable mortality rates involved applying the OAMFs to non-smoking plus non-alcohol-related mortality by stratum, instead of to all-cause mortality by stratum. For this purpose, we used previous estimates of both smoking-attributable mortality and alcohol--attributable mortality fractions, by year, sex, educational level (low, middle, high), and age (30+) (Van Hemelrijck et al. 2024; Van Hemelrijck et al. 2023). Subsequently, we estimated, by country, year, educational level, sex, and age, the share of mortality due to smoking and alcohol misuse combined, by means of multiplicative aggregation of the fractions for the two individual risk factors (Ezzati et al. 2003), using the following formula:

$$PAF_{\mathrm{sm\_alc}} = 1 - \prod_{\mathrm{i}=1}^{\mathrm{n}} (1 - PAF_{\mathrm{i}})$$

Where *PAF* stands for the population-attributable mortality fraction, *i* stands for the individual risk factor, and PAF_{sm_alc} stands for the population-attributable mortality fraction for smoking and alcohol misuse combined. Subsequently, we calculated non-smoking plus non-alcohol-related deaths by stratum, by multiplying – by stratum - the respective all-cause death numbers with 1-PAF_{sm_alc}.

To assess the yearly contributions of OAM to absolute educational inequalities in all-cause mortality, we used the Slope Index of Inequality (SII) as the main measure of absolute inequality in all-cause mortality. For this purpose, and similar to calculating the SII for obesity-attributable mortality, we first calculated the Relative Index of Inequality (RII). In calculating the RII we applied Poisson regression as per Moreno-Betancur et al. (2015) to the all-cause mortality data by educational level (low, middle, high), which includes the use of a multiplicative Poisson model by year and sex, adjusted for five-year age groups. The SII was calculated from the RII and the standardized death rate (SDR) in the general population (de Gelder et al. 2017):

$$\mathrm{SII} = \frac{2 * SDR * (RII - 1)}{(RII + 1)}$$

To obtain the standardised death rate, we applied – similarly to the obtainment of standardised obesity-attributable mortality - direct age standardisation using the 2013 revision of the European Standard Population (European Commission, 2013) as the standard population. This European Standard Population was based on the projected total (= male + female combined) population of the European Union (EU)-27 plus the European Free Trade Association (EFTA) countries, based on the Eurostat 2010-based population projections, averaged over the 2011-2030 period (European Commission, 2013). We applied this standard population for people aged 30 and over to the age-specific mortality rates by year, sex, and education group.

In addition, however, we also measured absolute inequality in all-cause mortality by the Rate Difference (RD), which is calculated by subtracting the standardized death rate for the high educated from the standardized death rate for the low educated (Mackenbach & Kunst, 1997).

Moreover, we assessed the yearly contributions of OAM to absolute educational inequalities in remaining life expectancy at age 30 (e30). For this purpose, we used two different inequality measures First, educational inequality in e30 measured by subtracting e30 for the low educated from e30 for the high educated (Zazueta-Borboa et al. 2023). Second, by the absolute Population Attributable Life Loss index, which is measured by subtracting e30 for all three educational groups combined from e30 for the high educated (Shkolnikov et al. 2001). The remaining life expectancy at age 30 by educational level and sex, which is used as input to both inequality measures, is calculated by applying standard demographic life table techniques (Preston et al. 2001) to age-specific mortality rates by country, year, sex, and educational level.

References

Costa, G. & Demaria, M. (1988) Un sistema longitudinale di sorveglianza della mortalità secondo le caratteristiche socio-economiche, come rilevate ai censimenti di popolazione: Descrizione e documentazione del sistema. *Epidemiologia e Prevenzione*, 36, 37–47.

Creeser, R. (2001). Turin Longitudinal Study, *Longitudinal Study Newsletter* 24, 2-3. London: Office of National Statistics and Centre for Longitudinal Studies, University of London, Institute of Education.

de Gelder, R., Menvielle, G., Kovács, K., Martikainen, P., Strand, B., & Mackenbach, J. P. (2017). Long-term trends of inequalities in mortality in 6 European countries. *International Journal of Public Health,* 62(1), 127-141. doi: 10.1007/s00038-016-0922-9

European Commission (2013) Revision of the European Standard Population — Report of Eurostat's task force. *EUROSTAT Methodologies and Working Papers.* Luxembourg: Publications Office of the European Union.

Gonzales Martinez, R. & Janssen, F. (2023) The creation of a database on obesity and high BMI prevalence by educational level and sex for adjacent calendar years and uniform ages for England, Finland, and Italy. *NIDI Working Paper* 2023/1. The Hague: Netherlands Interdisciplinary Demographic Institute.

Gonzales Martinez, R. & Janssen, F. (2024) Comparison of methods to estimate obesity-attributable mortality and high-BMI-attributable mortality by educational level in England & Wales, Finland, and Italy. *NIDI Working paper* 2024/1. The Hague: Netherlands Interdisciplinary Demographic Institute. 73 pages.

Hoffmann, R., Eikemo, T. A., Kulhánová, I., Kulik, M. C., Looman, C., Menvielle, G., ... & Mackenbach, J. P. (2015) Obesity and the potential reduction of social inequalities in mortality: evidence from 21 European populations. *The European Journal of Public Health*, 25(5), 849-856.

Janssen, F., Van Hemelrijck, W.M.J., Kagenaar, E., Sizer, A. (2024) Enabling the examination of long-term mortality trends by educational level for England and Wales in a time-consistent and internationally comparable manner. *Population Health Metrics,* 22(4), 1 - 19. doi: s12963-024-00324-2.

Kagenaar, E., Van Hemelrijck, W. M. J., Kunst, A. E. & F. Janssen (2022) Long-Term Trends in Obesity Prevalence by Socio-Economic Group in Five European Countries and the USA: The Relevance of the Diffusion of Innovations Theory. *Obesity Facts* ,15(6), 753-761. doi: 10.1159/000527070.

Lhachimi, S.K., Nusselder, W.J., Smit, H.A., van Baal, P., Baili, P., Bennett, K. et al. (2012) DYNAMO-HIA-A Dynamic Modeling Tool for Generic Health Impact Assessments. *Plos One* 7(5).

Lobstein, T., & Leach, R. J. (2010) *Workpackage 7: Overweight and obesity report on data collection for overweight and obesity prevalence and related relative risks.* https://webgate.ec.europa.eu/chafea_pdb/assets/files/pdb/2006116/2006116_d4_dynamo_hia.pdf

Mackenbach, J. P., & Kunst, A. E. (1997). Measuring the magnitude of socio-economic inequalities in health: An overview of available measures illustrated with two examples from Europe. *Social Science & Medicine,* 44(6), 757-771.

Moreno-Betancur, M., Latouche, A., Menvielle, G., Kunst, A.E., & Rey, G. (2015). Relative index of inequality and slope index of inequality: a structured regression framework for estimation. *Epidemiology,* 26(4). 518-527. doi: 10.1097/EDE.0000000000000311.

Office for National Statistics (2019) ONS Longitudinal Study – England and Wales. Released 11 June 2019, ONS SRS Metadata Catalogue. doi: 10.57906/z9xn-ng05.

Pascariu, M. D., Dańko, M. J., Schöley, J., & Rizzi, S. (2018) Ungroup: An R package for efficient estimation of smooth distributions from coarsely binned data, *Journal of Open Source Software,* 3(29), 937. doi:10.21105/joss.00937

Rizzi, S., Gampe, J., Eilers, P.H. (2015) Efficient estimation of smooth distributions from coarsely grouped data. *American Journal of Epidemiology,* 182, 138–147. doi: 10.1093/aje/kwv020.

Rizzi, S., Halekoh, U., Thinggaard, M., Engholm, G., Christensen, N., Johannesen, T.B., Lindahl-Jacobsen, R. (2019) How to estimate mortality trends from grouped vital statistics. *International Journal of Epidemiology,* 48, 571–582.

Shelton, N., Marshal, C.E., Stuchbury, R., Grundy, E., Dennett, A., Tomlinson, J., Duke-Williams, O., Xun, W. (2019) Cohort Profile: the Office for National Statistics Longitudinal Study (the LS). *International Journal of Epidemiology*, 48(2), 383–384. doi: 10.1093/ije/dyy243.

Shkolnikov, V., Valkonen, T., Begun, A., & Andreev, E.M. (2001). Measuring inter-group inequalities in length of life. *Genus*, LVII(3–4), 33–62.

The Global BMI Mortality Collaboration (2016) Body-mass index and all-cause mortality: individual-participant-data meta-analysis of 239 prospective studies in four continents. *The Lancet,* 388(10046), 776-786.

UNESCO (1997) *International Standard Classification of Education - ISCED 1997.* Paris: UNESCO Institute for Statistics.

Van Hemelrijck, W.M.J., Martikainen, P., Zengarini, N., Costa, G. & F. Janssen (2023). The impact of estimation methods for alcohol-attributable mortality on long-term trends for the general population and by educational level in Finland and Italy (Turin). *Plos One* 18(12): e0295760.

Van Hemelrijck, W., Kunst, A.E., Sizer, A., Martikainen, P., Zengarini, N., Costa, G. & F. Janssen (2024) Trends in educational inequalities in smoking-attributable mortality and their impact on changes in general mortality inequalities: evidence from England & Wales, Finland, and Italy (Turin). *Journal of Epidemiology & Community Health* 78: 561-569. doi: 10.1136/jech-2023-221702.

Vidra, N., Trias-Llimos, S., Janssen, F. (2019) Impact of obesity on life expectancy among different European countries: secondary analysis of population-level data over the 1975-2012 period. *BMJ Open,* July 31, 9(7), e028086. doi: 10.1136/bmjopen-2018-028086.

Zazueta-Borboa J.D., Martikainen, P., Aburto, J.M., Costa, G., Peltonen, R., Zengarini, N., Sizer, A., Kunst, A.E., Janssen, F. (2023) Reversals in past long-term trends in educational inequalities in life expectancy for selected European countries. *Journal of Epidemiology & Community Health,* 77, 421-429.

Appendix A1 - Underlying data sources obesity prevalence

- England => HSE (1991-2018)

Health Survey for England (HSE) - National Health Service (NHS) (UK Dataservice). http://content.digital.nhs.uk/healthsurveyengland ; www.ukdataservice.ac.uk/

- Finland => AVTK (1978-2014); EVTK (1993-2013, bi-annually); ATH (2015-2017); FinSote (2018-2020)

Health Behaviour and Health among the Finnish Adult Population (AVTK) – Finnish Institute for Health and Welfare (THL). https://http://www.thl.fi/en/web/thlfi-en/research-and-expertwork/population-studies; https://www.thl.fi/en/web/thlfi-en/statistics/information-for-researchers.

Health Behaviour and Health among the Finnish Retirement-Age Population (EVTK) – Finnish Institute for Health and Welfare (THL); https://thl.fi/fi/tutkimus-ja-kehittaminen/tutkimukset-ja-hankkeet/finsote-tutkimus/aiemmat-tutkimukset/elakeikaisen-vaeston-terveyskayttaytyminen-ja-terveys-evtk-.

Adult Health, Welfare and Service Research (ATH) - Finnish Institute for Health and Welfare (THL); https://thl.fi/fi/tutkimus-ja-kehittaminen/tutkimukset-ja-hankkeet/finsote-tutkimus/aiemmat-tutkimukset/aikuisten-terveys-hyvinvointi-ja-palvelututkimus-ath-

FinSote National Survey of Health - Finnish Institute for Health and Welfare (THL); https://thl.fi/en/web/thlfi-en/research-and-development/research-and-projects/national-finsote-survey

We obtained the data in aggregate format from our contact persons Suvi Parikka and Jonna Ikonen.

- Italy => NMSS (1990/1), HCHS (1994, 1999/2000, 2004/05, 2013), AVQ (2001-2003; 2005-2018)

All data from the National Multipurpose Social Survey (NMSS), Aspects of Daily Life (Aspetti della vita quotidiana) (AVQ) & Health Conditions and use of Health Services (HCHS) were obtained through the Italian National Institute of Statistics (ISTAT) (contact persons: Lidia Gargiulo and Antonella Ciccarese). The data from NMSS were obtained through the contact persons in aggregate format, and the deidentified individual HCHS data ((http://www.istat.it/it/archivio/5471) were obtained through the contact persons. The deidentified individual data from AVQ http://www.istat.it/it/archivio/4630 were partly obtained through the contact persons, and partly through the ISTAT website: https://www.istat.it/it/archivio/186843 (2005-2012) and http://www.istat.it/it/archivio/129956 (2013-2018).
