# Supplementary material for: Trends in educational inequalities in obesity‐attributable mortality in England and Wales, Finland, and Italy
Source: Obesity (Silver Spring). 2025 Feb 18;33(3):578–88. doi: 10.1002/oby.24225 (PMC11897850; doi:10.1002/oby.24225)
Supplement: Supplementary file 2 — Supplementary File S2: Supplementary Tables and Figures. [file OBY-33-578-s002.pdf]

TRENDS IN EDUCATIONAL INEQUALITIES IN OBESITY-ATTRIBUTABLE MORTALITY IN ENGLAND & WALES, FINLAND, AND ITALY

SUPPLEMENTARY FILE 2 – SUPPLEMENTARY TABLES AND FIGURES

Figure S1 - Trends in relative educational inequalities in obesity-attributable mortality, measured by the Relative Inequality Index (RII), by sex and country, for people aged 30 and older in England & Wales (1991-2017), Finland (1978-2017), and Italy (Turin)(1990-2018)

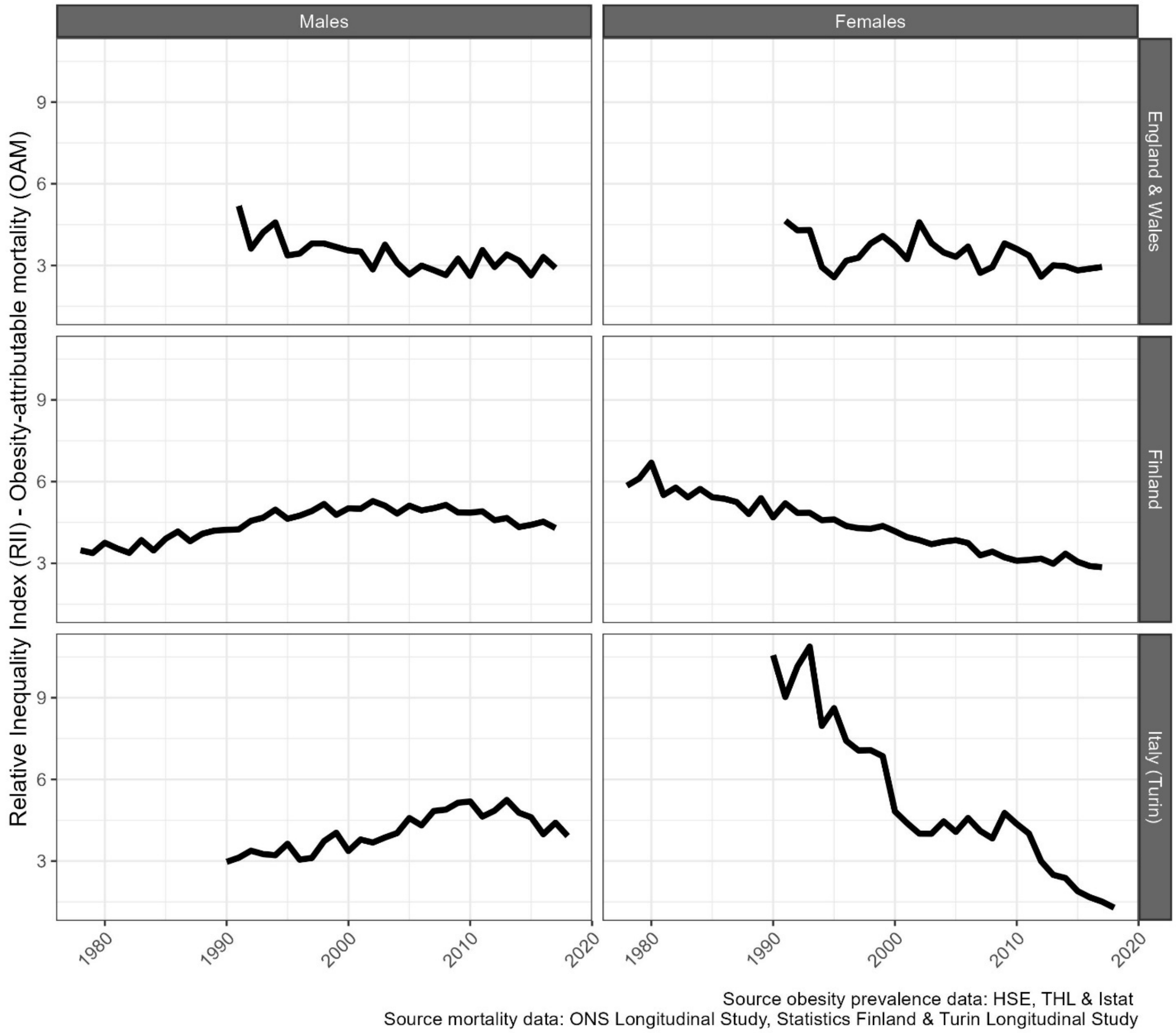

Figure S2 - The (changing) relative contribution of obesity-attributable mortality to educational inequalities in all-cause mortality or remaining life expectancy, based on different inequality measures, for people aged 30 and older, by sex and country, in England & Wales (1991-2017), Finland (1978-2017), and Italy (Turin)(1990-2018)

a) **Contribution of OAM to the rate difference (RD) in standardised mortality (30+)**

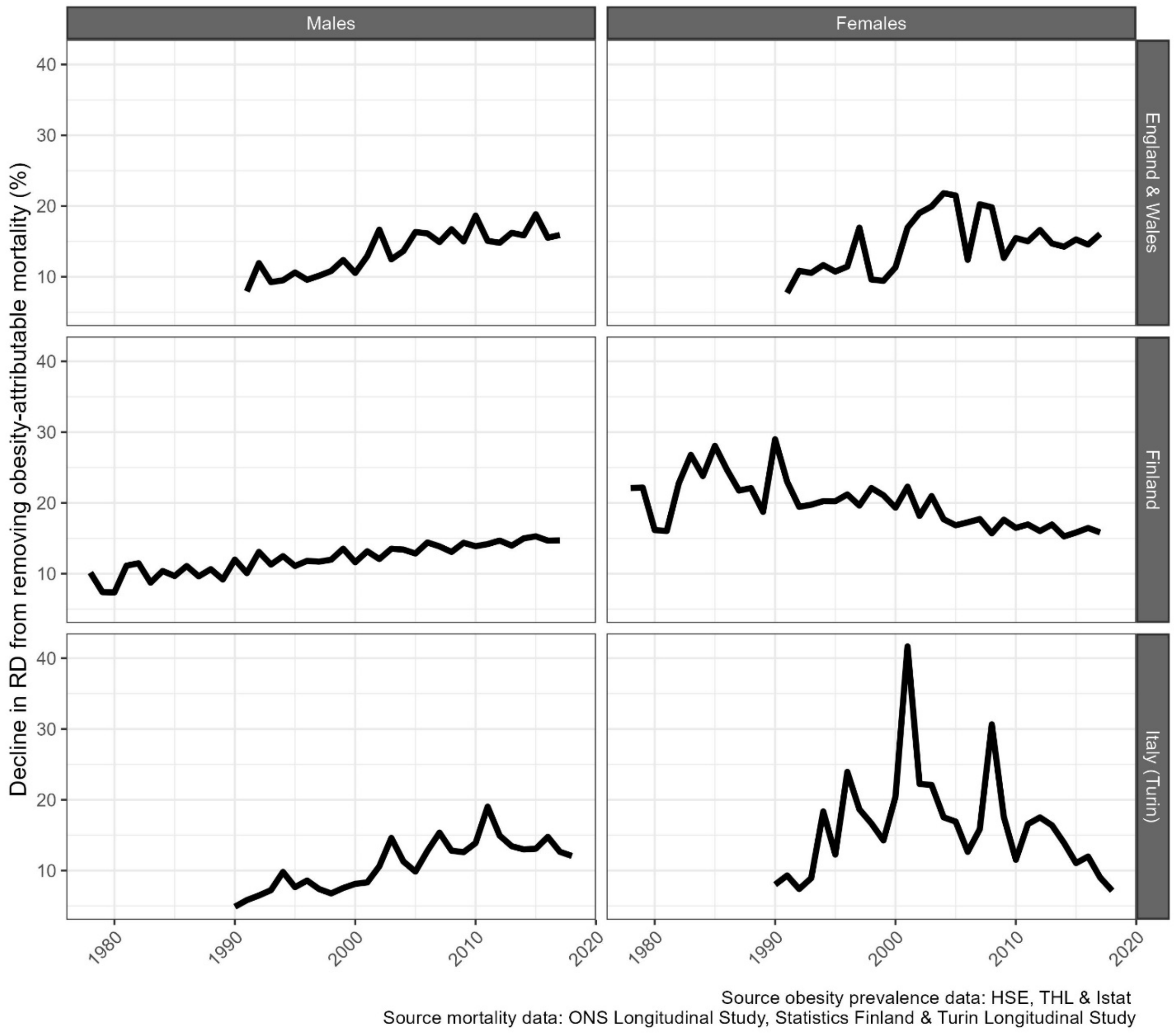

b) Contribution of OAM to educational inequalities in e30 (e30_ineq)(measured by e30 high educated minus e30 low educated)

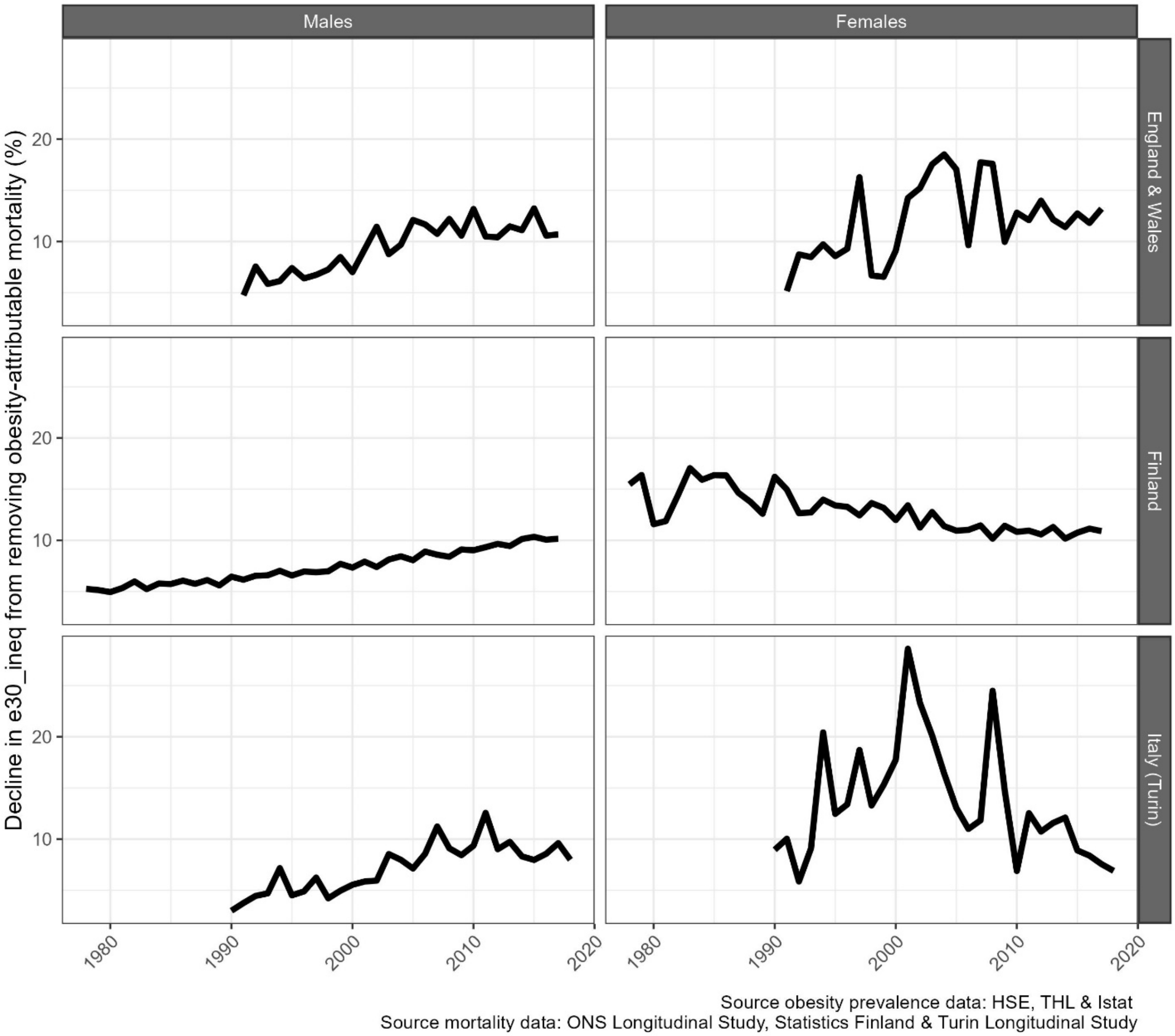

c) Contribution of OAM to the absolute Population Attributable Life Loss index (PALLabs) (measured by e30 high educated minus e30 for the total population)

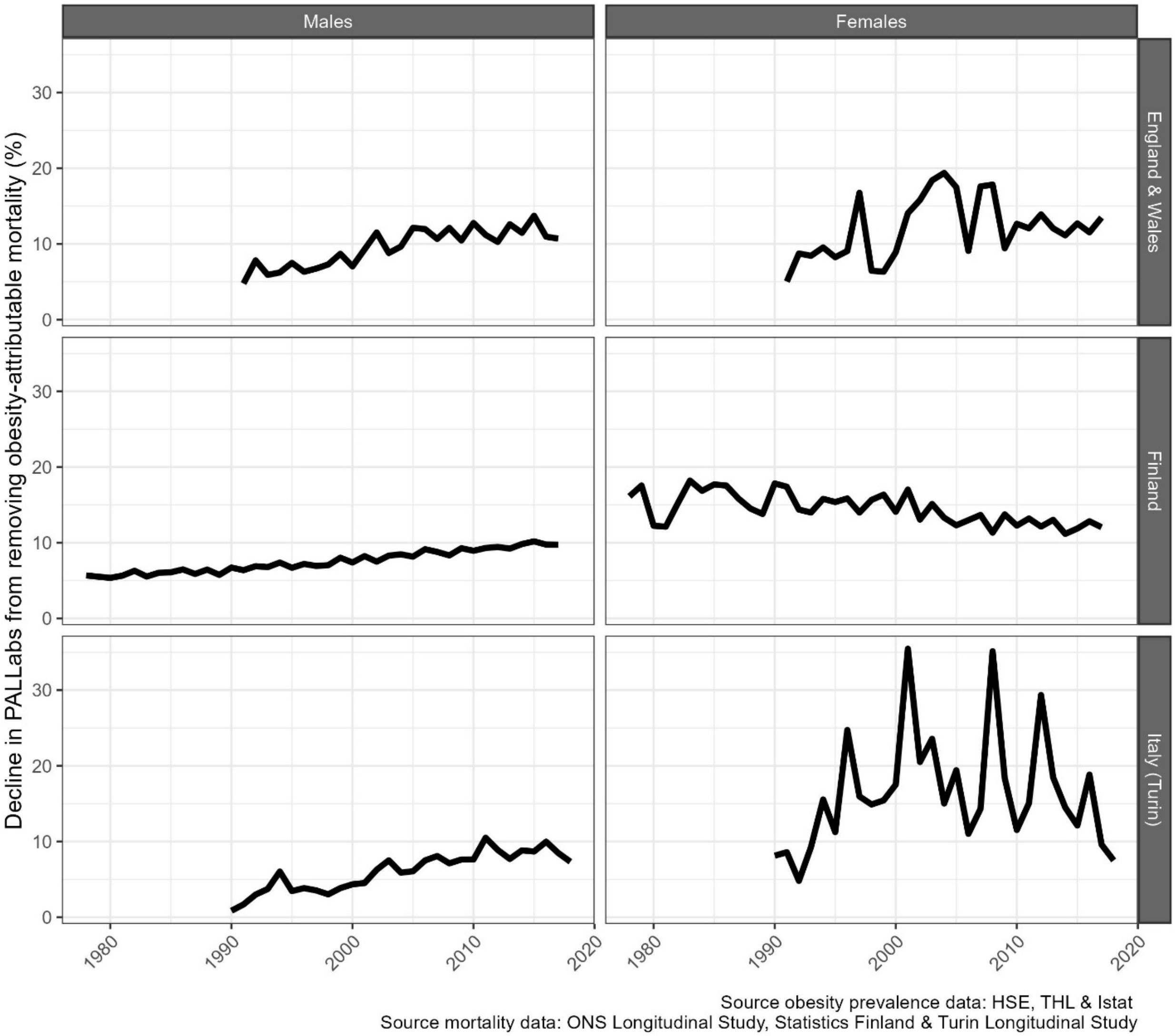

Figure S3 - Trends in standardized obesity-attributable mortality rates – estimated by applying the fractions to non-smoking & non-alcohol-attributable mortality – by educational level, by sex and country, for people aged 30 and older in England & Wales (1991-2017), Finland (1987-2017)*, and Italy (Turin) (1990-2018)

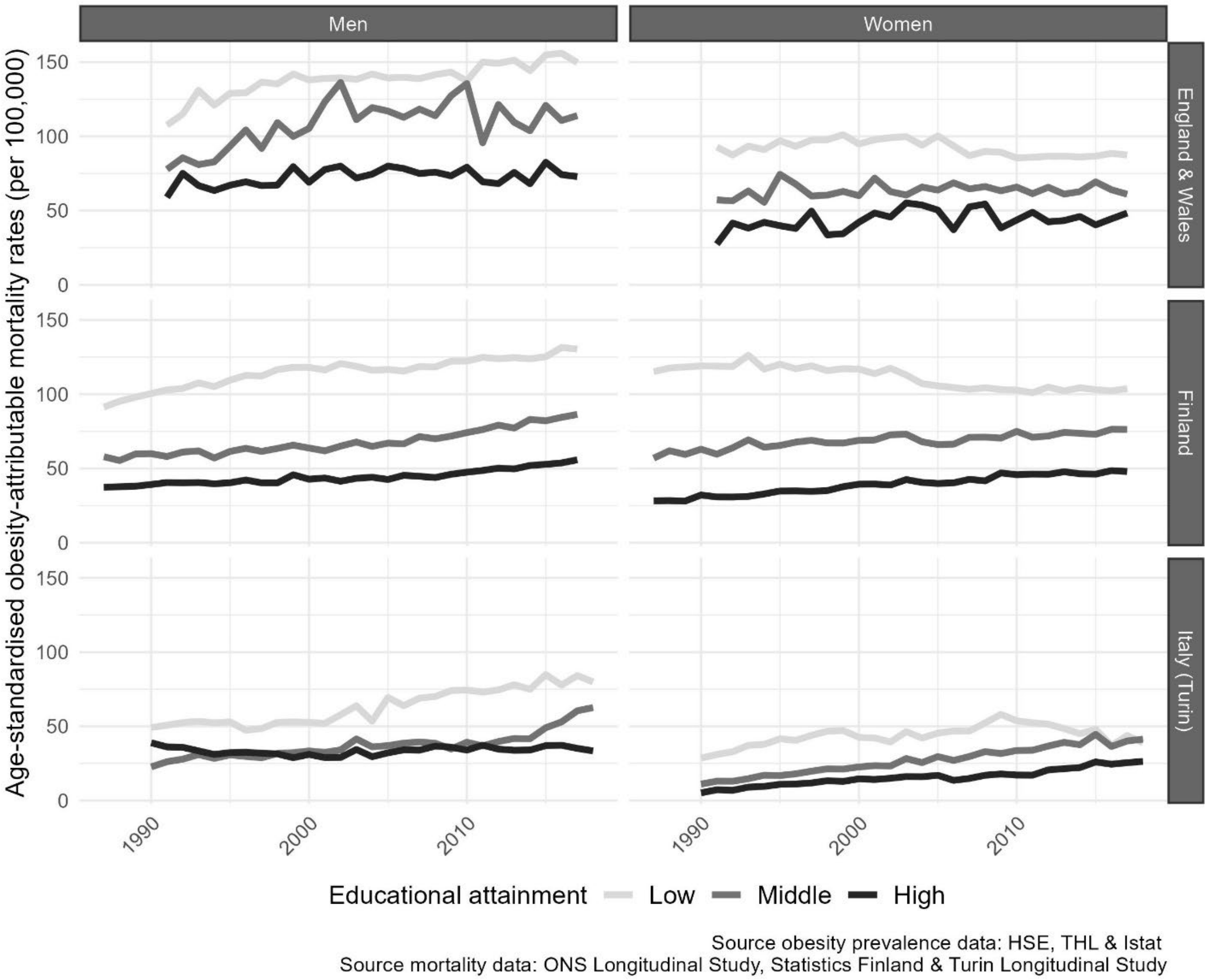

* For Finland, we have estimates for alcohol-attributable mortality only from 1987 onwards

Figure S4 – Trends in standardized obesity prevalences by educational level, by sex and country, for people aged 30 and older in England (1991-2017), Finland (1978-2017), and Italy (1990-2018)

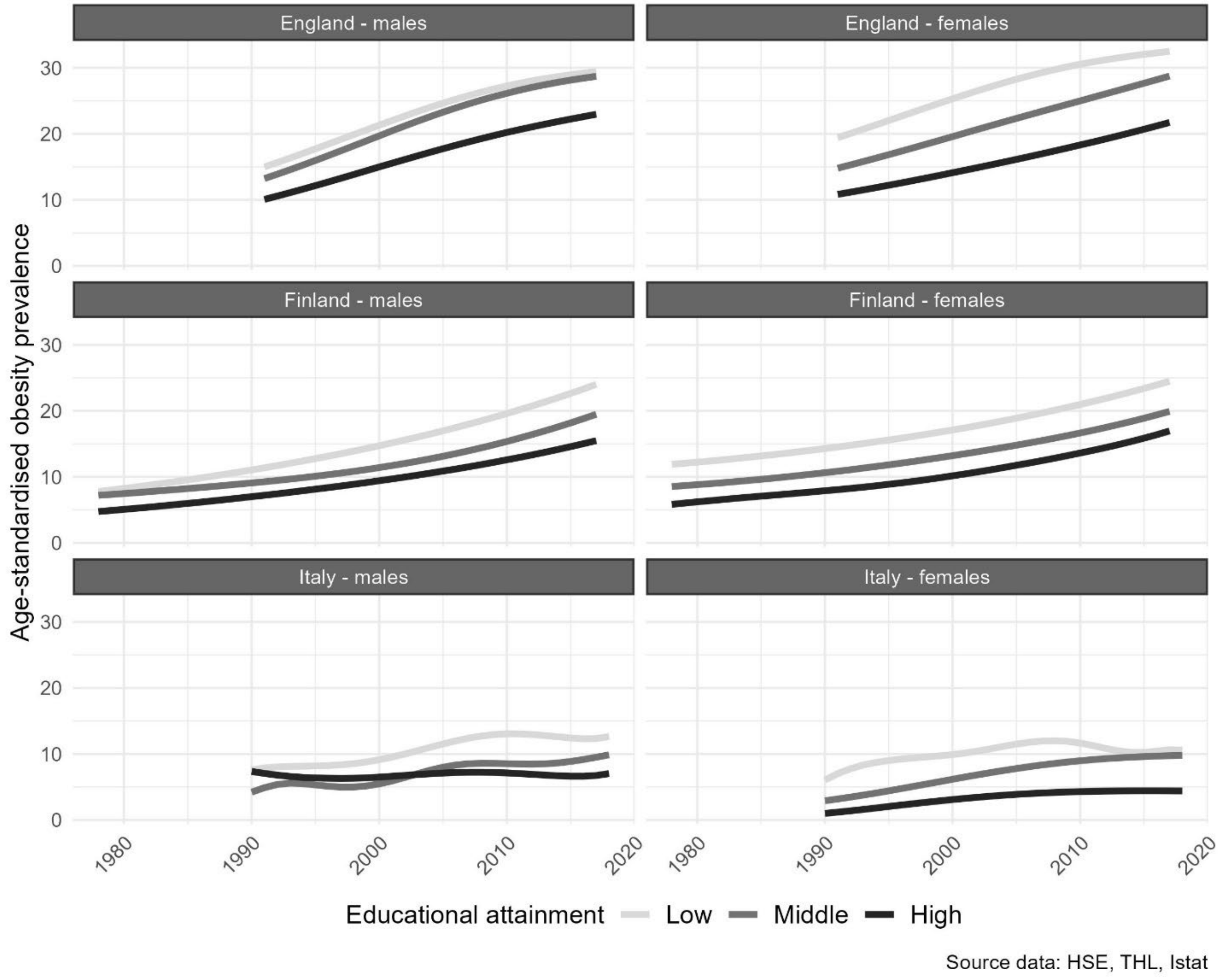

Table S1 – Absolute and relative contribution of obesity-attributable mortality to absolute educational inequalities in all-cause mortality or remaining life expectancy, based on different inequality measures, in 1991, 2017, and averaged over 1991-2017, for people aged 30 and older, by sex and country

	Absolute contribution			Relative contribution		
Population	1991	2017	1991-2017	1991	2017	1991-2017
Decline in the Slope Index of Inequality (SII) from removing obesity-attributable mortality (absolute: per 100,000; relative: %)						
England & Wales - Males	211.5	162.4	185.4	7.0	15.6	12.8
England & Wales - Females	146.0	101.9	125.8	9.0	14.6	14.0
Finland - Males	173.1	177.8	192.9	8.5	13.3	11.3
Finland - Females	158.6	92.0	124.3	17.6	15.4	16.9
Italy (Turin) - Males	76.6	106.8	93.8	8.2	12.0	12.0
Italy (Turin) - Females	54.6	17.7	53.6	15.1	7.0	21.0
Average (unweighted)	136.7	109.8	129.3	10.9	13.0	14.7
Average - males	153.7	149.0	157.4	7.9	13.7	12.1
Average - females	119.7	70.6	101.2	13.9	12.3	17.3
Decline in Rate Difference (RD) from removing obesity-attributable mortality (absolute: per 100,000; relative: %)						
England & Wales - Males	96.4	113.2	100.9	7.9	15.9	13.6
England & Wales - Females	82.4	66.6	68.8	7.7	16.1	14.7
Finland - Males	92.9	114.4	109.8	10.1	14.7	13.2
Finland - Females	91.1	71.7	79.5	23.0	15.8	18.5
Italy (Turin) - Males	30.9	75.5	54.8	5.8	12.7	11.1
Italy (Turin) - Females	27.9	19.8	33.9	9.3	9.0	16.9
Average (unweighted)	70.3	76.9	74.6	10.6	14.0	14.7
Average - males	73.4	101.1	88.5	8.0	14.4	12.6
Average - females	67.1	52.7	60.7	13.3	13.6	16.7
Decline in educational inequality in remaining life expectancy at age 30 (e30) (e30 high educated minus e30 low educated) from removing obesity-attributable mortality (absolute: in years; relative: %)						
England & Wales - Males	0.3	0.6	0.5	4.7	10.7	9.4
England & Wales - Females	0.5	0.6	0.5	5.1	13.2	12.1
Finland - Males	0.3	0.7	0.5	6.1	10.2	8.2
Finland - Females	0.4	0.5	0.5	15.0	10.9	11.9
Italy (Turin) - Males	0.1	0.4	0.3	3.8	9.6	7.3
Italy (Turin) - Females	0.2	0.2	0.3	10.0	7.6	14.0
Average (unweighted)	0.3	0.5	0.4	7.5	10.3	10.5
Average - males	0.3	0.5	0.4	4.9	10.1	8.3
Average - females	0.4	0.4	0.4	10.0	10.5	12.7
Decline in the Population Attributable Life Loss index (e30 high educated minus e30 overall) from removing obesity-attributable mortality (absolute: in years; relative: %)						
England & Wales - Males	0.3	0.4	0.4	4.8	10.7	9.6
England & Wales - Females	0.4	0.4	0.4	5.0	13.5	12.1
Finland - Males	0.3	0.3	0.3	6.4	9.7	8.3
Finland - Females	0.4	0.3	0.3	17.4	12.0	13.8
Italy (Turin) - Males	0.0	0.2	0.1	1.7	8.5	6.2
Italy (Turin) - Females	0.2	0.1	0.2	8.6	9.6	17.0
Average (unweighted)	0.3	0.3	0.3	7.3	10.7	11.2
Average - males	0.2	0.3	0.3	4.3	9.6	8.0
Average - females	0.3	0.3	0.3	10.3	11.7	14.3

Source obesity prevalence data: HSE, THL & Istat

Source mortality data: ONS Longitudinal Study, Statistics Finland & Turin Longitudinal Study

Table S2 – The contribution of obesity to educational inequalities in all-cause mortality, comparison of the results by Hoffmann et al. 2015 to our results, by sex and country, 2001-2006

	Hoffmann et al. 2015 (ages 30-79)			Our results for 2001-2006 (unweighted averages) (ages 30+)					
				SII			RD		
Population	initial RD	decline RD	decline ineq (%)	ineq allcause	decline ineq	decline ineq (%)	ineq allcause	decline ineq	decline ineq (%)
England & Wales - Males	410	23	5.6	1418.5	188.4	13.3	689.4	99.2	14.7
England & Wales - Females	245	30	12.2	934.7	142.2	15.2	380.5	67.3	18.6
Finland - Males	615	15	2.4	1777.2	199.9	11.2	844.0	111.4	13.2
Finland - Females	260	20	7.7	746.2	125.4	16.8	417.2	78.1	18.9
Italy (Turin) - Males	304	30	9.9	832.8	93.0	11.2	494.2	54.5	11.2
Italy (Turin) - Females	51	21	41.2	264.8	52.7	19.9	178.1	33.8	22.2
Average (unweighted)	314.2	23.2	13.2	995.7	133.6	14.6	500.6	74.0	16.5
Average - males	443.0	22.7	6.0	1342.9	160.4	11.9	675.9	88.4	13.1
Average - females	185.3	23.7	20.4	648.6	106.7	17.3	325.3	59.7	19.9

Source of our obesity prevalence data: HSE, THL & Istat
Source of our mortality data: ONS Longitudinal Study, Statistics Finland & Turin Longitudinal Study

Note: Differences in the population-specific contributions of Hoffmann et al. 2015 can most likely be explained by differences in the underlying data and in the methodology used.
Hoffmann et al. 2015 used the Finbalt Health Monitor (94/98/00/02/04) to obtain prevalence data for Finland, which covers obesity prevalence data for the adult Finnish population (30-64), whereas we used both the yearly AVTK data (30-64) and the bi-annual EVTK data (65-84) and interpolation plus smoothing techniques to obtain yearly data for people aged 30 and older. For Italy (Turin), Hoffmann et al. 2015 relied on the national prevalence data (30-79) from the HCHS in 1999-2000, whereas we used the data from the HCHS in 1999-2000 combined with the yearly national prevalence data from the AVQ (from 2001 onwards) to obtain smoothed yearly prevalence data for people aged 30 and older. For England & Wales, Hoffmann et al. 2015 relied – similar to us – on the HSE data, but only for a single year (2001) and for people aged 30-79, whereas we used yearly data from the HSE (1991-2018; 30-85+), which we smoothed by age and over time.
In addition, the data regarding all-cause mortality by educational level in England & Wales might be slightly different because we adjusted the data for trend discontinuities related to data issues, and for differences with country-level mortality data for the total population (Janssen et al. 2024).
Moreover, the estimates by Hoffmann et al. (2015) stem from the effect of reducing the obesity prevalence around the year 2000 among the least educated groups to that of the highest educated group on relative all-cause mortality inequalities measured by the Rate Ratio for 21 European populations around 2001-2006. By contrast, we assessed at the population level the full potential effect of eliminating obesity-attributable mortality for the three different educational groups on absolute educational inequalities in mortality (measured by the SII), thereby applying standardization to obtain comparable outcomes.
